# 417. Should We Be Doing This? Evaluating Pleural Fluid Inoculation into Blood Culture Bottles

**DOI:** 10.1093/ofid/ofae631.131

**Published:** 2025-01-29

**Authors:** J Hunter Fraker, Matthew S Lee

**Affiliations:** Beth Israel Deaconess Medical Center, Boston, MA; Beth Israel Deaconess Medical Center, Boston, MA

## Abstract

**Background:**

Sterile fluid inoculation into blood culture (BCx) bottles has primarily been shown to be beneficial with peritoneal fluid. However, we have observed widespread expansion of this practice for other sterile fluid specimens at our institution. We investigated whether there was a clinical benefit to inoculating pleural fluid specimens into BCx bottles.

**Figure:**
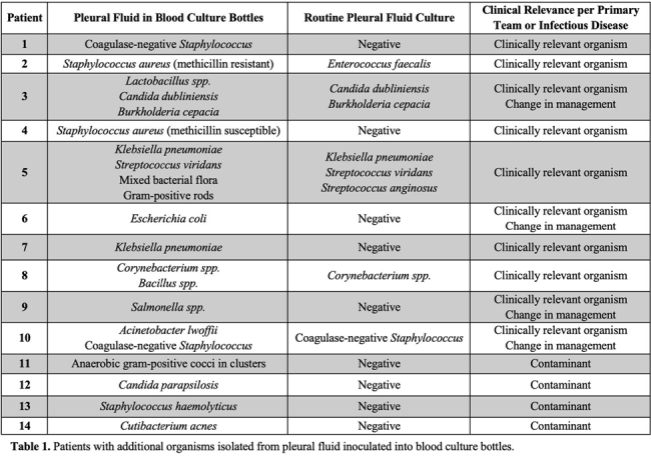

**Methods:**

This was a retrospective chart review of patients who had pleural fluid inoculated into BCx bottles from July 1, 2021 through June 30, 2023. Clinical, microbiologic, and pharmacy data were extracted from medical records of patients that had at least one organism isolated from these samples and had a routine (non-blood culture bottle inoculation) pleural fluid culture sent from the same procedure. The primary outcome was the proportion of clinically relevant organisms based on evaluation by the primary or a consultant team.

**Results:**

A total of 334 pleural fluid specimens were inoculated into BCx bottles during the study period; positive cultures were found in 12.2% (41/334). Five patients were excluded as they did not have a concomitant routine pleural fluid culture, leaving a study cohort of 36 patients.

In the study cohort (n=36), 61.1% (22/36) had the same organism(s) isolated from both BCx inoculated and routine pleural fluid cultures, while 38.9% (14/36) had additional organisms from the BCx bottle inoculated specimens that were not isolated on the routine pleural fluid culture.

Of the 14 patients with additional organisms from BCx bottle inoculation (Table 1), 4 were deemed contaminants. Ten patients had clinically relevant organisms and 4 of these led to changes in antimicrobial management (change in agent or duration).

Overall, 3% (10/334) of specimens with pleural fluid inoculated into BCx bottles had a clinically relevant organism that was not isolated in routine culture. 1.2% (4/334) of specimens led to a change in clinical management based on the additional organism(s) isolated.

**Conclusion:**

In our single-institution study, we found widespread practice of inoculating pleural fluid into blood culture bottles. The practice may slightly increase microbiological yield over routine fluid cultures, but few patients had a change in clinical management at our institution.

**Disclosures:**

**All Authors**: No reported disclosures

